# AI-Mediated Informal Digital Learning of English for Speaking Development: Longitudinal Effects on Ability and Affect and Implications for Adaptive Intelligence

**DOI:** 10.3390/jintelligence14090204

**Published:** 2026-09-01

**Authors:** Difei Jia, Xi Chen, Yunsong Wang

**Affiliations:** 1 School of Foreign Languages, North China University of Water Resources and Electric Power, Zhengzhou 450046, China; jdf@ncwu.edu.cn; 2 School of Foreign Languages and Cultures, Nanjing Normal University, Nanjing 210023, China

**Keywords:** AI-mediated informal digital learning of English (AI-IDLE), adaptive intelligence, speaking ability, speaking anxiety, speaking enjoyment

## Abstract

Generative artificial intelligence (GenAI) has expanded opportunities for English learning beyond formal classroom settings. From an intelligence perspective, speaking in English as a foreign language (EFL) represents an applied domain in which learners must adaptively retrieve, integrate, monitor, and deploy linguistic knowledge under communicative demands. The present study examined the effects of a pedagogically guided form of AI-mediated informal digital learning of English (AI-IDLE) on Chinese university EFL learners’ speaking ability, speaking anxiety, and speaking enjoyment. To this end, 89 Chinese university EFL learners participated in a 12-week intervention and were assigned to an experimental group (EG) or a control group (CG). Pre- and post-intervention evaluations were administered to assess the results, including standardized speaking skills and valid questionnaires regarding enjoyment and speaking anxiety. The English-speaking skills test and the two questionnaires were administered at post-test and again 10 weeks after the intervention as the delayed post-test. Mixed-effects modeling (MEM) was used, and the findings showed that the EG demonstrated significantly greater improvements in speaking ability and enjoyment and a greater reduction in speaking anxiety than the CG, with these between-group advantages evident at the post-test. Overall, the findings suggest that structured AI-IDLE can provide a digitally mediated context for intelligence-relevant adaptive learning by supporting communicative performance while fostering affective conditions conducive to its development. These findings have implications for understanding how digitally mediated informal learning environments may support adaptive intelligence in applied language-learning contexts.

## 1. Introduction

Developing EFL learners’ speaking skills has long been a major challenge in language education because gaining the needed communication abilities has always stimulated people to follow language learning (Kirkpatrick et al., 2025; Peng & Wang, 2024; R. Zhao, 2024). Within the four language skills, speaking is a core contributor to communication within the target language and forms students’ general language development (Q. Li & Chan, 2024; Wan & Moorhouse, 2024). Contrary to receptive proficiencies, speaking needs quick language retrieval and voluntary production, which usually makes it challenging for students to achieve correct and confident performance. Inadequate chances for interaction within the EFL setting made students typically feel doubtful and anxious to communicate in public (Papi & Khajavy, 2023; J. Yang & Yang, 2025; Zhou et al., 2023). Speaking anxiety is a form of anxiety related to emotional feelings of apprehension, worry, and discontent when speaking in a foreign language (R. Zhao, 2024). Among other issues, this fear-like emotion comes along with physiological alterations like enhanced respiration and heart rate, muscular trembling, and over-reactive behaviors (Nadeem et al., 2025). Speaking anxiety is recognized as one of the core impediments in language learning when learning a foreign language (Hawanti & Zubaydulloevna, 2023). This type of anxiety can lower students’ participation, enhance avoidance behaviors, and adversely influence speaking skill growth.

On the contrary, there has been a recent increase in research on positive emotions within the language learning field (e.g., Bensalem et al., 2025; Derakhshan & MacIntyre, 2025, 2026; Derakhshan & Park, 2026; Dewaele & MacIntyre, 2024; Dewaele et al., 2023; Paradowski & Jelińska, 2024; Pham & Tin, 2022; Y. Wang, 2026; Y. Wang & Gao, 2026; Zuniga, 2023). This is especially the case for enjoyment (e.g., Y. Jiang & Dewaele, 2019; Jin et al., 2025). In the wider PP structure of language teaching, Foreign Language Enjoyment is found to be a vital element in successful language learning (Gao et al., 2024; C. Li, 2020). Speaking enjoyment indicates a positive emotional condition that shows satisfaction, confidence, and interest within speaking communication tasks (Yetkin & Aslan, 2026). Students who experience enjoyment potentially become engaged dynamically in speaking activities, enhance their speaking performance over time, and maintain effort (Zhang et al., 2024). Taken together, negative emotions like anxiety can confine the absorption of language input; however, positive emotions like enjoyment boost learners’ consciousness regarding language input (Dewaele & Dewaele, 2020). Moreover, there is a small negative correlation between anxiety and enjoyment, though these two aspects seem to be autonomous emotions rather than opposite ends of an identical aspect (Dewaele & MacIntyre, 2014).

Therefore, improving EFL students’ speaking skills, enhancing their enjoyment, and lowering their anxiety is a permanent challenge (Fernández-García & Fonseca-Mora, 2022). To address these issues, an increasing body of literature indicates that by offering automatic feedback and assessment, AI provides creative chances for speaking practice (Fathi et al., 2024; Solhi et al., 2026; Zou et al., 2023). AI technologies offer personalized learning routes and immediate feedback (L. Yang, 2026; Zhang et al., 2024). Language teachers and scholars are increasingly supporting generative artificial intelligence (GenAI) integration tools into language learning, especially as interactive communicative peers (Fu et al., 2026; Hwang & Chen, 2023; Y. Wang & Guo, 2026; Zou et al., 2023). GenAI shows revolutionary progress in AI that develops new materials, offers contextual answers based on natural language processing, and engages in human-like conversation (Paul et al., 2023). In EFL contexts, GenAI is different from traditional AI tools thanks to its flexibility, interactivity, and offering of customized feedback (Derakhshan, 2025; Derakhshan & Li, 2026). Indeed, GenAI provides L2 students with increased linguistic input and increased chances for meaningful interaction, consistent with interactionist theory (Ji et al., 2023; C. Wang et al., 2025). ChatGPT, as a GenAI, attracted scholars’ attention thanks to its progressive natural language processing capabilities (Han, 2024; R. Wu & Yu, 2024). Its capability to generate text and comprehend can develop language learning through active and relevant communications that endure a natural communicative flow (Peng & Liang, 2025). Recent scholarship has conceptualized the use of AI tools for learner-directed English learning beyond formal classroom settings as AI-mediated informal digital learning of English (AI-IDLE) (Liu & Lee, 2026). AI-IDLE foregrounds human–AI interaction and learners’ active engagement with AI resources outside conventional classroom instruction. Because the ChatGPT speaking activities were pedagogically guided but completed outside regular class time and allowed learners flexibility in pacing, topic exploration, and interaction, they are treated as a pedagogically guided form of AI-IDLE rather than fully autonomous informal learning. However, comparatively little longitudinal intervention research has examined whether AI-IDLE-oriented speaking practice can simultaneously influence speaking ability, speaking anxiety, and speaking enjoyment and whether these intervention-associated advantages remain evident after the immediate intervention period.

## 2. Review of the Literature

### 2.1. Theoretical Framework

Within the AI-IDLE framing described above, Self-Determination Theory (SDT; Ryan & Deci, 2020) is used as an interpretive lens for understanding learners’ performance-related and affective experiences during AI-mediated speaking practice. SDT proposes that learning environments can shape motivation and emotional experience through the psychological needs for autonomy, competence, and relatedness. From an SDT perspective, several affordances of GenAI-mediated speaking practice may be relevant to the psychological needs for autonomy, competence, and relatedness (Chiu, 2024). The flexibility to practice at one’s own pace and explore topics of personal interest may create conditions supportive of autonomy, while immediate feedback and adaptive responses may be relevant to learners’ perceived competence. Similarly, the responsive and nonjudgmental nature of AI-mediated interaction may provide a degree of communicative support that can be considered in relation to relatedness. From an SDT perspective, these affordances may provide a useful theoretical explanation for why AI-mediated speaking practice could be associated with improved speaking performance and more positive affective experiences. However, because autonomy, competence, and relatedness were not directly measured in the present study, SDT is used here as an interpretive framework rather than as an empirically tested mediational mechanism.

### 2.2. Speaking Proficiency

Developing speaking is broadly known as one of the most challenging proficiencies for language students, specifically within the EFL settings in which chances for speaking interaction within the target language are limited (Z. Chen, 2024; Derakhshan & Fathi, 2024). Speaking ability can be understood as the capacity to produce comprehensible spoken language that fulfills communicative purposes and enables effective interaction with both native and non-native speakers in authentic contexts (Q. Li & Chan, 2024; Wan & Moorhouse, 2024). Instead of being a separate skill, speaking has a core effect in forming general language growth (H. Wu, 2025). In this respect, AI-based tools can develop responsive and interactive settings that support the practice and development of speaking proficiency (Jeon, 2024; Y. Wang et al., 2026; Yuan & Liu, 2025). These tools are specifically beneficial for EFL students because they provide chances consistent with efficient educational practices, like customized and quick feedback on fluency, pronunciation, and other dimensions of speaking performance (R. Jiang, 2022). In this way, by offering personalized feedback and maintaining chances for repeated practice, GenAI tools can deal with a number of the restrictions of conventional language instruction. Accordingly, more research is suggested to precisely examine the educational effect of GenAI within language teaching on EFL students’ speaking results.

### 2.3. Speaking Enjoyment

Regarding learning psychology in L2, enjoyment was found to be a core positive emotion, including a sense of self-satisfaction, interest, pride, and pleasure that take place when students perceive linguistic learning or advancement (X. Chen & Li, 2025). This emotion is usually felt when students feel mentally provoked, socially supported, and able to successfully engage with language activities (Peng & Wang, 2024). Within a language learning setting, enjoyment motivates students to investigate language utilization with higher freedom, more in-depth engagement in communicative activities, and to persevere despite linguistic difficulties (Song, 2024). In EFL settings, enjoyment is related to maintained communicative engagement, enhanced motivation, and more adaptive responses to the inherent difficulties of language learning (Bensalem et al., 2026; Peng & Wang, 2024). Moreover, enjoyment has a vital effect in forming students’ responses to the difficulties embedded in speaking communication. Speaking in a foreign language usually includes ambiguity, time pressure, and fear of making mistakes; nevertheless, students experiencing enjoyment potentially adaptively respond by persevering in communication and looking for chances to enhance their performance (Solhi & Derakhshan, 2026). In this regard, enjoyment acts as a positive emotional condition and a cognitive and motivational source that maintains students’ long-term engagement with learning a language (Derakhshan & Yin, 2025; H. Wu, 2025). The growing incorporation of online tools in language teaching has allowed GenAI tools like conversational chatbots to offer stress-free and interactive settings in which students can exercise speaking, engage in meaningful conversation, and receive quick feedback (Gao & Quan, 2025). These characteristics may contribute to more positive speaking experiences and greater confidence and, from an SDT perspective, may also provide conditions relevant to learners’ sense of autonomy during speaking tasks. Thus, investigating the effect of GenAI-based settings on EFL students’ enjoyment is vital for developing our comprehension of the way new technologies can support the communicative and emotional aspects of language development.

### 2.4. Speaking Anxiety

The sense of fear and anxiety in FL communication is speaking anxiety, which is known as an important obstacle to successful language learning (R. Zhao, 2024). This type of anxiety can impede students’ ability to initiate immediate communication, lead to lower participation in language interactions, and reduce the quality of verbal answers, inhibiting their advancement in language proficiency (MacIntyre & Gregersen, 2022). Students suffering from speaking anxiety do not tend to express themselves due to fears of error commitment (Z. Chen, 2024). This apprehension can enhance speaking anxiety and therefore restrain speaking capability. Indeed, speaking anxiety affects learners’ presentation and their perceptions of communicative competence and classroom experience. To overcome speaking anxiety, it seems necessary to establish a supportive and low-risk learning setting where students feel secure mentally to communicate without fear of excessive correction (X. Wang & Wen, 2025). Promoting such a cohesive and relaxing setting is vital for students to overcome speaking anxiety and gain greater performance. In this respect, recent studies have investigated the possibility of technology-based teaching to reduce anxiety. For example, in a review of 28 empirical studies, D. Li and Zhao (2025) investigated the efficiency of AI-based tools in improving students’ speaking communication. Their results showed that AI-based apps can considerably enhance students’ confidence by offering quick feedback, personalized practice, and decreased social tension, enhance speaking fluency, and decrease anxiety levels. Nevertheless, empirical investigations that concurrently investigate speaking anxiety besides speaking performance and enjoyment in GenAI-based teaching settings are limited. Therefore, the present study attempts to explain the way new GenAI tools can influence EFL students’ speaking anxiety and improve speaking performance and enjoyment.

### 2.5. Current Study

Recent studies (Z. Chen, 2024; X. Chen & Li, 2025; R. Zhao, 2024) suggest that Chinese EFL learners continue to experience difficulties in spoken English, including problems with intonation and pronunciation as well as limited confidence in oral communication. These difficulties are both linguistic and psychological. Grounded in the broaden-and-build theory (Fredrickson, 2004) and the control-value theory (Pekrun, 2024), based on research, language students usually experience varying emotional conditions within speaking activities, which can negatively or positively affect their performance (Derakhshan & Yin, 2025; Dewaele et al., 2023). Challenges in speaking are usually followed by elevated anxiety and decreased enjoyment in communication. Therefore, studying the emotional elements like enjoyment and anxiety in speaking has both a theoretical value and a necessary practical importance. The present research focused on anxiety and enjoyment because of their interconnected effects in speaking performance (Yuan & Liu, 2025; R. Zhao, 2024). Directed by a positive sense, enjoyment improves motivation and participation, while anxiety prohibits progress, resulting in decreased communication and avoidance. Considering their important effect on speaking, intervention studies were conducted to improve positive emotions and reduce negative ones (e.g., Zhang et al., 2024; Yetkin & Aslan, 2026). Indeed, AI, and particularly GenAI, provide a promising answer to this difficulty due to their ability for human-resembling interaction and personalized feedback. For instance, in a systematic review of 24 studies, Du and Daniel (2024) investigated the effect of AI technologies for speaking practice, and the research underscored some benefits like improved learning through customized and interactive experiences, enhanced pronunciation through quick feedback, improved motivation and engagement because of the interactive essence of AI tools, and decreased speaking anxiety because of a supportive and secure setting. Moreover, in a study conducted by Zhang et al. (2024), the effect of an AI-speaking instruction on Chinese EFL learners’ willingness to communicate (WTC), anxiety, and enjoyment was investigated. The findings emphasize the effectiveness of AI-speaking tools in improving EFL learners’ WTC and FLE while lowering FLA.

Similarly, Marpaung (2024) studied the impact of Gemini AI on speaking skills, and the results revealed an improvement in speaking skills, confidence, vocabulary, and fluency. Gemini, as a GenAI, led to higher speaking performance. Similarly, Zou et al. (2023) studied the viewpoints of EFL learners on AI tools used for practicing speaking, indicating that the use of AI enhanced their speaking skills. Students underscored the facilitation of using AI, stating that it promoted an autonomous and flexible learning experience. In their study, Tai and Chen (2024) researched the effect of GAI chatbots on the speaking skills of EFL students, concentrating on two interaction layouts, paired and individual. Based on the findings, both types of interactions with GenAI showed positive influences, and the participants felt enjoyment, motivation, and engagement regarding chatbot-based EFL speaking. Moreover, GenAI developed a supportive setting and rapport, which improved their confidence and decreased speaking anxiety. In a study performed by He et al. (2025), the effect of the GenAI voice chatbot on enhancing students’ speaking skill development was investigated. The research underscored the multimodal essence of the chatbot, incorporating speech recognition and interactive communication to offer students an authentic conversation exercise and natural language articulation. The findings showed that providing quick feedback and adaptive structure in speaking tasks through GenAI improves students’ tendency to communicate, confidence, and speaking fluency. In a study conducted by X. Wang and Wen (2025), the effect of AI tools in improving speaking proficiency, enhancing motivation, and decreasing speaking anxiety of EFL students was investigated. The research findings indicated that students using AI tools showed significantly higher growth in speaking competence and reduced speaking anxiety when they had higher motivation. The ability of AI tools in lowering learners’ speaking anxiety was also investigated by Nadeem et al. (2025). Based on the findings, AI tools can offer a non-judgmental and relaxed environment, enhancing learners’ confidence and reducing speaking anxiety. The effect of GenAI on learners’ English-speaking skills, linguistic risk-taking (LRT), and speaking anxiety was investigated by Y. Chen et al. (2025). Based on the findings, GenAI enhanced speaking skills and LRT, along with a significant decrease in anxiety. These results offer information on the efficiency of GenAI in improving learners’ English-speaking learning results and emotional activities.

Despite the rapid development of AI-mediated informal language learning research, several gaps remain. Recent research on AI-mediated language learning has expanded rapidly across speaking, oral communication, and affective outcomes (Ji et al., 2023; D. Li & Zhao, 2025; Zhang et al., 2024). Within the more specific AI-IDLE literature, a recent scoping review indicates that the field remains dominated by cross-sectional research and that longitudinal intervention evidence is still comparatively limited (Liu & Zhao, 2026). Prior AI-IDLE research has also highlighted the role of motivation and enjoyment (Liu et al., 2024), while enjoyment and anxiety have been identified as important affective dimensions of AI-mediated informal language learning (X. Zhao et al., 2026). Nevertheless, fewer longitudinal intervention studies have examined whether structured out-of-class AI-mediated speaking practice can simultaneously support speaking ability, reduce speaking anxiety, increase speaking enjoyment, and show evidence of retention at follow-up. The present study addresses this gap by examining a pedagogically guided form of AI-IDLE among Chinese university EFL learners. As a result, these research questions were formulated:

RQ1: What is the effect of AI-mediated informal digital speaking practice on EFL learners’ speaking ability, speaking anxiety, and speaking enjoyment compared with conventional speaking activities over time?

RQ2: To what extent do the intervention-associated advantages of AI-mediated informal digital speaking practice remain evident at the 10-week delayed post-test?

By examining speaking ability, speaking enjoyment, and speaking anxiety within the same longitudinal design, the present study extends AI-IDLE research beyond its predominant focus on learner engagement, antecedents, and cross-sectional associations. In particular, the study investigates whether a pedagogically guided form of out-of-class AI-IDLE can support both performance-related and affective dimensions of speaking development and whether these advantages remain evident ten weeks after the intervention. Theoretically, SDT is used as an interpretive lens for considering the affordances of AI-mediated informal speaking practice, while the psychological mechanisms proposed by SDT are not directly tested. Pedagogically, the study provides evidence concerning how structured AI-mediated informal speaking practice may supplement conventional EFL speaking instruction.

## 3. Method

### 3.1. Participants

Participants within the present research included 89 EFL students in China who registered in English courses in a Chinese college. Convenience sampling was used to select the learners because they were available to the scholars and were in the same speaking-oriented English course within the semester where the research was performed. The participants ranged in age from 18 to 22 years and represented different academic majors. Although some participants reported occasional personal use of AI tools, they had limited experience using GenAI as part of a structured classroom learning environment. This characteristic helped establish a relatively comparable starting point for examining the instructional effects of the intervention. The study adopted a quasi-experimental longitudinal design. Participants were recruited within the same speaking-oriented course context rather than from multiple independently sampled instructional settings. Following convenience recruitment, the 89 participants were allocated to an experimental group (EG; *n* = 45) or a control group (CG; *n* = 44). Because individual random assignment was not employed, baseline equivalence between the two groups was examined statistically before the intervention. The former received speaking instruction through GenAI tools, while the latter followed conventional speaking activities based on the regular curriculum. Independent-samples comparisons at pretest indicated no statistically significant group differences in speaking ability, speaking anxiety, or speaking enjoyment, suggesting comparable baseline levels on the study outcomes.

All 89 participants completed the pretest, post-test, and delayed post-test assessments. Consequently, participant retention was 100%, and no attrition occurred during the 10-week follow-up period (EG: 45/45; CG: 44/44). Because no participants withdrew from the study or had missing outcome data at any measurement occasion, no imputation procedures were required. All longitudinal analyses were therefore conducted using complete-case data and included all participants in their originally assigned groups throughout the study. The absence of participant attrition minimized the potential for attrition bias and strengthened the internal validity of the longitudinal comparisons.

### 3.2. Instruments

#### 3.2.1. First Certificate in English Test (Speaking)

To guarantee students’ agreement considering language proficiencies, the FCE exam speaking section was chosen from Cambridge English Language Assessment (2015), which is a standardized exam bank. This exam assesses students’ speaking skills and includes four parts in which they need to effectively talk in in-person settings. The exam takes 14 min, during which students need to answer questions and become engaged in an English dialogue. The initial part includes a conversation between the manager and each learner (speaking questions); however, the next part comprises learners’ lengthy turns for individual volunteers, with a short answer from another volunteer (offered a pair of pictures for debate). The next part comprises a two-way communication among learners seeking to reach a decision, and the ultimate part concentrates on a debate regarding the subjects dealt with in Part 3 (speaking questions). Importantly, communications within the oral section took place with classmates and other volunteers, taking almost 14 min. To reduce potential practice effects across repeated assessments, parallel speaking task sets were selected from the same standardized FCE task bank for the pretest, post-test, and delayed post-test. The three sets followed the same four-part format and comparable task structure, while the specific questions, pictures, and discussion prompts were rotated across measurement occasions so that participants were not repeatedly exposed to identical speaking tasks. An administrator questions learners while another one hears them and writes them down. Speaking performance was evaluated using four analytic dimensions: grammar and vocabulary, discourse management, pronunciation, and interactive communication. Each dimension was rated on a 0–5 scale, yielding a composite speaking score ranging from 0 to 20. Two trained raters evaluated the speaking performances independently after a calibration session in which they reviewed the scoring criteria and discussed sample performances. The ratings were then combined to obtain the final speaking score used in the subsequent analyses. Inter-rater reliability was assessed using an intraclass correlation coefficient (ICC) based on the two raters’ scores, indicating satisfactory agreement between the two raters (ICC = 0.92).

#### 3.2.2. Speaking Enjoyment Questionnaire

Speaking enjoyment was assessed using a five-item measure adapted from the enjoyment scale employed by P. Zhao et al. (2023). Because the original items focused on L2 writing enjoyment, the wording was adapted to refer specifically to English speaking while preserving the original meaning of the items. A sample item is “I feel very happy when I am speaking English.” The five items were treated as a unidimensional measure of speaking enjoyment and were rated on a 5-point Likert scale ranging from 1 (“strongly disagree”) to 5 (“strongly agree”), yielding a possible total score ranging from 5 to 25, with higher scores indicating greater speaking enjoyment. Internal consistency was satisfactory across all three measurement occasions (Cronbach’s α = 0.86, 0.89, and 0.88 for the pretest, post-test, and delayed post-test, respectively).

#### 3.2.3. Speaking Anxiety Questionnaire

In the present research, an adapted version of the anxiety questionnaire developed by Öztürk and Gürbüz (2014) was utilized, in which they selected 18 items from among 33 FLCAS items. It deals with the most common source of FL speaking anxiety. The questionnaire was based on a Likert scale from 1 (strongly disagree) to 5 (strongly agree). The reliability of the scale was 0.92, 0.93, and 0.92 across the three measurement occasions in this study.

### 3.3. Procedure

At the beginning, the researcher informed the participants of the study objectives and distributed the consent forms to the participants. To protect participants’ privacy, all collected data were anonymized and used solely for research purposes. Ethical procedures were followed throughout the study in accordance with the institutional guidelines governing research involving human participants. In addition, the participants filled in a background scale to offer demographic information and specifications considering their English learning history and their familiarity with AI, a pretest to evaluate their primary speaking skill, and the enjoyment and speaking anxiety questionnaire. At the beginning of the semester, 89 Chinese EFL learners were recruited and allocated to either the EG (*n* = 45) or the CG (*n* = 44) within the quasi-experimental design. Within the intervention, participants engaged in ChatGPT-based tasks developed to enhance their speaking skills. A primary orientation session offered instruction on utilizing ChatGPT through voice control of the ChatGPT tool (Version GPT-4o) for voice-based interactions. Within these interactions, ChatGPT played the role of a peer by asking follow-up questions, encouraging clarifications, proposing further explanations, and offering immediate feedback. To improve consistency across learners, all participants followed the same general interaction protocol. At the beginning of each session, learners were instructed to ask ChatGPT to act as an English-speaking partner on the assigned topic, ask one question at a time, encourage elaboration, and provide brief feedback after each completed response. A typical prompt was: “Act as my English-speaking partner on this topic. Ask me one question at a time, encourage me to explain my ideas, and after I finish each response, provide brief feedback on pronunciation, grammar, vocabulary, and communicative clarity.” Learners were encouraged to complete their responses before receiving corrective feedback so that the interaction retained a conversational flow. Feedback focused primarily on salient errors that affected accuracy or comprehensibility rather than correcting every error. Each session ended after approximately 15 min with a brief summary of recurring language problems and suggestions for improvement. The same ChatGPT voice interface and task instructions were used throughout the intervention. Because ChatGPT responses may vary dynamically, standardization was implemented primarily through the common task structure, prompt instructions, feedback criteria, and session duration rather than through identical AI-generated responses. The intervention lasted for 12 weeks. During this period, learners in the EG engaged in AI-mediated informal digital speaking practice twice a week outside regular class time, with each session lasting approximately 15 min. Thus, each learner completed approximately 24 sessions, corresponding to about 360 min (6 h) of AI-mediated speaking practice over the intervention period. The subjects of the speaking tasks were consistent with the material of the English book. Participants were encouraged to draw on classroom learning while also exploring topics of personal interest, allowing the out-of-class activities to combine pedagogical guidance with learner-directed AI-mediated informal digital learning. Intervention participation was monitored through weekly learning logs submitted via Forms. Learners recorded the frequency and approximate duration of their ChatGPT speaking sessions, the topics discussed, and brief reflections on the interaction and feedback received. The logs were reviewed regularly to monitor whether learners were following the expected twice-weekly practice schedule and whether their activities remained aligned with the assigned speaking themes. Participants were reminded when records were incomplete or when the reported practice deviated from the expected procedure. The logs therefore served as a practical fidelity check, although platform-level system usage logs were not available.

In contrast, participants within the CG received conventional speaking teaching based on the common class syllabus with no use of GenAI tools. The speaking exercise included teacher-instructed communicative tasks like pair debates and role-plays. The speaking exercise for the CG was developed to be compared in duration and frequency to that of the EG; however, interactions took place with partners instead of with a GenAI peer. The CG completed conventional speaking activities with approximately equivalent frequency and practice duration, including pair discussions and role-plays, but without GenAI-mediated interaction. Following the intervention, post-tests were administered to assess the changes in speaking proficiency, speaking anxiety, and enjoyment. To investigate the durability of the instructional effects, a delayed post-test was conducted ten weeks after the completion of the intervention. The resulting longitudinal dataset, consisting of pretest, post-test, and delayed-post-test measurements, was subsequently analyzed using mixed-effects modeling to examine changes in speaking ability, speaking anxiety, and speaking enjoyment associated with AI-mediated informal digital speaking practice over time.

### 3.4. Data Analysis

The collected data were analyzed using IBM SPSS Statistics Version 27. Prior to the main analyses, the dataset was screened for accuracy, missing values, and potential outliers. The reliability of the speaking enjoyment and the speaking anxiety questionnaires was evaluated using Cronbach’s alpha coefficients. In addition, independent-samples *t*-tests were conducted on the pretest scores to examine whether both groups were comparable before the intervention. To investigate the changes in three variables across the three measurement occasions, mixed-effects modeling was employed. This analytical approach was selected because the study involved repeated observations nested within individuals over time. For each dependent variable, a linear mixed-effects model was estimated with group (EG vs. CG), time (pretest, post-test, and delayed post-test), and the Group × Time interaction specified as fixed effects. Repeated observations were nested within participants, and participant-specific random intercepts were included to account for dependency among repeated measurements. Model coefficients were estimated using restricted maximum likelihood (REML). To examine the appropriateness of the random-effects structure, random-intercept models were compared with models additionally allowing random slopes for time using maximum-likelihood estimation. Across the three outcomes, the more parsimonious random-intercept models showed lower AIC and BIC values and were therefore retained. Residual distributions and plots of residuals against fitted values were also inspected, and no substantial departures from normality or homoscedasticity were identified. Participants were drawn from the same speaking-oriented course context and the intervention was implemented within the same instructional setting; therefore, no additional class- or instructor-level random effect was specified. The main effect of time was examined to determine overall changes across the study period, whereas the main effect of group indicated whether the two groups differed in their overall performance. The group × time interaction served as the primary indicator of treatment effectiveness because it revealed whether the EG demonstrated a different developmental pattern from that of the CG. The first research question was addressed by examining the fixed-effect estimates associated with group, time, and the group × time interaction for speaking ability, speaking anxiety, and speaking enjoyment. The second research question focused on the sustainability of the intervention effects and was examined through comparisons involving the delayed post-test scores. Estimated marginal means and Bonferroni-adjusted pairwise comparisons were used to identify the specific occasions on which significant differences occurred within and between groups. Statistical significance was determined using an alpha level of 0.05. Also, parameter estimates and confidence intervals were reported to facilitate a clearer interpretation of the magnitude and direction of the observed effects, which enabled a comprehensive examination of both the immediate and sustained effects of GenAI tools on Chinese EFL learners’ speaking performance, anxiety, and enjoyment. Longitudinal changes in speaking ability, speaking anxiety, and speaking enjoyment were examined using separate linear mixed-effects models. Because these three outcomes represent theoretically distinct dimensions of L2 speaking development (performance, affective barriers, and positive emotional engagement), separate models were estimated rather than combining them into a single multivariate model. The analyses were guided by theoretically specified research questions focusing on each outcome independently. Although multiple dependent variables were examined, we did not apply an additional Bonferroni correction across the three mixed-effects models because such an adjustment would substantially increase the likelihood of Type II errors and potentially obscure meaningful intervention effects across conceptually different constructs. Instead, statistical significance was interpreted alongside confidence intervals, effect sizes, and the consistency of the observed interaction patterns across outcomes.

## 4. Results

Prior to addressing the research questions, the reliability of the study instruments was examined. Cronbach’s alpha coefficients indicated satisfactory internal consistency for both the speaking enjoyment and anxiety questionnaires across the three measurement occasions. The obtained values exceeded the minimum recommended threshold of 0.70, suggesting that the instruments were appropriate for subsequent analyses.

As shown in Table 1, the speaking enjoyment questionnaire demonstrated good internal consistency, with alpha coefficients ranging from 0.86 to 0.89. Similarly, the speaking anxiety questionnaire showed excellent reliability across the three administrations, with alpha values ranging from 0.92 to 0.93. These findings indicate that both instruments produced stable and reliable measurements throughout the study.

Independent-samples *t*-tests in Table 2 revealed no significant differences between the two groups on speaking ability, speaking anxiety, or speaking enjoyment at the pretest stage (all *p* > .05). These results indicated no statistically significant baseline differences between the two groups on the three measured outcomes (all *p*s > .05).

Inspection of the descriptive statistics in Table 3 revealed improvements in both groups over time. Participants in the EG showed higher speaking ability and speaking enjoyment scores and lower speaking anxiety scores at both the post-test and delayed post-test. The CG also demonstrated modest improvement across the three outcome variables, indicating that conventional speaking practice and general course-related factors contributed to learning gains. Importantly, the delayed-post-test descriptive statistics indicate that the experimental group continued to outperform the control group across all three outcomes 10 weeks after the intervention. Specifically, the experimental group maintained higher speaking ability (15.31 vs. 11.48), lower speaking anxiety (46.57 vs. 58.46), and higher speaking enjoyment (20.54 vs. 16.21) than the control group, suggesting that the intervention-associated benefits were retained over time. Furthermore, the significant Group × Time interaction terms confirmed that these sustained advantages exceeded the improvements attributable to general instructional exposure alone.

The mixed-effects analyses were based on participant-specific random-intercept models, allowing baseline differences among participants to be modeled while estimating the fixed effects of group, time, and their interaction.

The mixed-effects analysis in Table 4 revealed significant Group × Time interaction effects for both the post-test and delayed post-test assessments. These findings indicate that the EG improved significantly more than the control group in speaking ability over time. To facilitate interpretation of the practical significance of these differences, standardized effect sizes were also calculated for the between-group comparisons. At the post-test, the difference in speaking ability between the experimental and control groups corresponded to a very large standardized effect (Hedges’ *g* = 2.22; approximately Cohen’s *d* = 2.24). A similarly large effect remained evident at the delayed post-test (Hedges’ *g* = 2.02), indicating that the observed between-group difference was not only statistically significant but also educationally meaningful. The positive interaction coefficients therefore suggest that the benefits of GenAI-integrated instruction remained evident throughout the 10-week follow-up period.

Significant interaction effects in Table 5 emerged for speaking anxiety. Students in the EG exhibited a substantially greater anxiety reduction than those in the control group. The between-group standardized effect sizes were also very large (Hedges’ *g* = −1.97 at the post-test and −1.80 at the delayed post-test), confirming that the reduction in speaking anxiety was substantial in practical as well as statistical terms. Although a slight increase in anxiety occurred between the post-test and delayed post-test, anxiety levels remained considerably lower than baseline levels.

The results in Table 6 demonstrated significant interaction effects for speaking enjoyment. Learners exposed to GenAI-integrated instruction reported substantially greater enjoyment than those in the control group. The standardized effect sizes likewise indicated very large intervention effects (Hedges’ *g* = 2.28 at the post-test and 2.02 at the delayed post-test), demonstrating that the observed differences were educationally meaningful in addition to being statistically significant. Although a slight decline was observed at the delayed post-test, enjoyment levels remained considerably higher than pre-intervention scores.

Bonferroni-adjusted comparisons in Table 7 indicated significant improvements from pretest to post-test and from pretest to delayed post-test across all outcome variables. No statistically significant differences emerged between post-test and delayed-post-test scores. Taken together with the significant improvements relative to baseline, these findings suggest that substantial intervention-associated gains remained evident at the 10-week follow-up. The mixed-effects analyses provided consistent evidence of more favorable changes in the experimental group than in the control group across the three outcomes. Relative to conventional instruction, the GenAI-mediated condition was associated with greater gains in speaking ability and enjoyment and greater reductions in speaking anxiety. Importantly, these findings were not interpreted solely based on statistical significance; the consistency of the Group × Time interaction effects across three theoretically distinct outcomes, together with the large standardized effect sizes, provided converging evidence for the robustness and educational relevance of the intervention effects. Furthermore, direct between-group comparisons at the delayed post-test demonstrated that the experimental group maintained superior speaking ability (15.31 vs. 11.48), lower speaking anxiety (46.57 vs. 58.46), and higher speaking enjoyment (20.54 vs. 16.21) than the control group. These findings, together with the significant Group × Delayed interaction effects observed in the mixed-effects models, indicate that the advantages associated with GenAI-integrated instruction were sustained throughout the 10-week follow-up period. Because the follow-up interval was limited to 10 weeks, these findings should be interpreted as evidence of medium-term retention rather than long-term effectiveness. The standardized between-group effect sizes are summarized in Table 8.

## 5. Discussion

The present study examined longitudinal differences between AI-mediated and conventional speaking practice in EFL learners’ speaking ability, speaking anxiety, and speaking enjoyment. It is important to note that the control group also showed improvement from pretest to post-test across the three outcomes. These changes suggest that regular speaking instruction and repeated participation in speaking activities were themselves beneficial. General study-related influences, such as repeated practice, greater familiarity with the assessment procedures, or participation-related effects, may also have contributed to improvement over time. Accordingly, the specific advantage associated with the AI-mediated condition is more appropriately represented by the significant Group × Time interactions, which indicate improvement beyond the changes observed under conventional speaking instruction. The mixed-effects modeling analysis revealed significantly more favorable changes in speaking performance in the GenAI-mediated condition than in the conventional speaking condition over time. The findings therefore provide evidence that structured AI-mediated speaking practice was associated with greater speaking development relative to the active comparison condition, which is in line with Zhang et al. (2024), emphasizing that AI tools typically incorporate interactive elements, like communicative elements and immediate corrections, which are particularly beneficial for improving speaking. The tools imitate authentic language use, which enables learners to practice and receive quick feedback in AI-based settings. Similarly, the findings support those of Tai and Chen (2024), arguing that EFL students enjoyed English learning and showed greater WTC, enhanced communicative confidence, and lowered anxiety within the less risky setting developed by Google Assistant. From an SDT perspective, these results may be tentatively interpreted in relation to the affordances of AI-mediated environments for autonomy, competence, and relatedness. For example, adaptive and immediate feedback may represent an affordance relevant to perceived competence, while opportunities to control the pace of practice and experiment with language may be considered relevant to autonomy. Similarly, the responsive nature of interaction with an AI peer may provide a limited form of communicative support that can be discussed in relation to relatedness. These interpretations concern theoretically relevant affordances of the learning environment rather than empirically demonstrated satisfaction of the three psychological needs. The results of the present research are consistent with Y. Chen et al. (2025), indicating that GenAI results in better speaking ability among learners. Viewed through an AI-IDLE perspective, this finding is also consistent with broader evidence linking informal digital language learning to L2 achievement (Liu & Soyoof, 2026). The present study extends this line of research by examining structured out-of-class AI-mediated speaking practice across repeated measurement occasions. The present study adds to this literature by showing that structured out-of-class AI-mediated speaking practice may be particularly relevant to speaking development over repeated measurement occasions.

The findings provide evidence that the GenAI-mediated condition was associated with greater improvement in EFL learners’ speaking enjoyment across the intervention period, suggesting that the GenAI-supported activities may positively influence learners’ engagement with speaking tasks. The results showed that enjoyment was enhanced when GenAI was incorporated into speaking tasks. This pattern is consistent with emerging research showing that enjoyment and related affective experiences are important dimensions of learners’ engagement with AI-mediated English learning and speaking practice (Liu et al., 2024; Zhang et al., 2024; X. Zhao et al., 2026). In this sense, the present findings extend earlier AI-IDLE research by linking enjoyment not only to participation in informal learning but also to a structured speaking intervention over time. This enhancement can be related to GenAI’s ability to offer different and customized interactions suitable for students’ educational needs and individual interests, giving more enjoyment and meaning to the speaking activities, which is consistent with Yetkin and Aslan (2026). From an SDT perspective, learners’ control over the frequency, content, and pace of speaking practice may be interpreted as an autonomy-relevant affordance of AI-mediated learning. Customized feedback and opportunities for repeated interaction may likewise represent features theoretically relevant to perceived competence and communicative support. These affordances may help explain the more positive affective experiences observed in the AI-mediated condition. However, because autonomy, competence, and relatedness were not directly measured, these interpretations should be understood as theoretically plausible explanations rather than evidence of psychological need satisfaction. In addition, the low-stress settings developed by GenAI can decrease students’ apprehension of negative assessment and encourage more enjoyable speaking experiences, which is consistent with the study of Tai and Chen (2024). Students’ enjoyment can be enhanced, promoting higher engagement in speaking practice as they achieve confidence through frequent interactions with GenAI. These results are consistent with those of Zhang et al. (2024), who argue that by offering supportive and interactive situations for language practice, AI-based language learning settings can improve students’ enjoyment.

The findings also indicate that the GenAI-mediated condition was associated with a greater reduction in EFL learners’ speaking anxiety over time. Based on the results, GenAI offers a secure and non-threatening setting that enables students to get engaged in speaking activities with no typical apprehension of criticism or embarrassment. Contrary to human interlocutors, whose answers can accidentally lead to social tension, GenAI’s immediate and neutral behavior enabled learners to adjust their speaking speed, generate thoughts without the apprehension of perceived impatience or ridicule, and accept linguistic risks. This privacy and predictability may potentially reduce the physiological symptoms of anxiety, like a quick heartbeat and nervousness, that repeatedly impede automatic generation in conventional EFL settings. Therefore, GenAI acts as a stress-free practice peer that particularly lowers the fear of negative social assessment. Technology allows learners, specifically those who struggle in conventional class settings, to get engaged in speaking activities with higher confidence and ease by providing a psychologically secure setting. These findings may also be interpreted cautiously from an SDT perspective. The control afforded by GenAI Voice Mode over speaking pace, content, and timing may represent an autonomy-relevant feature of the learning environment. Similarly, frequent low-risk opportunities for practice and feedback may be theoretically relevant to perceived competence, while the responsive nature of AI interaction may provide a degree of communicative support. Such affordances offer possible explanations for the observed reduction in speaking anxiety, but they should not be interpreted as evidence that learners’ needs for autonomy, competence, or relatedness were actually satisfied. The results of the present research are consistent with those of Nadeem et al. (2025), emphasizing the motivational dimensions of AI tools, mentioning that learners have less anxiety and higher engagement while working with non-human communicative peers. The results were strongly consistent with those of X. Wang and Wen (2025), stressing the emotional dimensions of AI tools. The research indicated that students who use AI tools experience reduced anxiety levels and higher motivation, generally because of the unbiased features of AI partners. These results are consistent with those of Y. Chen et al. (2025), suggesting that by developing a stress-free and supportive setting for language practice, GenAI-based interaction could help reduce speaking anxiety.

## 6. Conclusions and Implications

The present study indicates that a pedagogically guided form of AI-IDLE was associated with greater improvements in speaking ability and enjoyment and greater reductions in speaking anxiety than time-matched conventional speaking activities. These findings suggest that structured out-of-class AI-mediated speaking practice can provide a useful supplementary pathway for EFL speaking development. Rather than demonstrating the absolute or isolated value of GenAI, the findings show a relative advantage of the AI-mediated informal speaking condition over the active conventional comparison condition. More broadly, the study extends AI-IDLE research by jointly examining a performance-related outcome and two affective outcomes within a longitudinal intervention design. From a theoretical perspective, SDT offers a useful interpretive lens for understanding the observed findings. The flexibility of AI-mediated speaking practice may be relevant to learners’ autonomy, while repeated low-risk practice and immediate feedback may be relevant to perceived competence. The responsive nature of the interaction may also provide a degree of communicative support. However, because the three basic psychological needs were not directly measured, the present study does not establish that changes in autonomy, competence, or relatedness mediated the effects on speaking ability, anxiety, or enjoyment. These SDT-based interpretations should therefore be regarded as theoretically plausible explanations that require direct empirical testing in future research. In relation to intelligence research, the study therefore suggests that AI-IDLE may function as an applied digital context for supporting and examining adaptive, intelligence-relevant performance, while anxiety and enjoyment represent important affective conditions under which such performance develops.

The findings also offer several practical implications for AI-mediated informal digital speaking practice. Rather than relying on occasional or unstructured use of GenAI, teachers may incorporate brief but regular out-of-class speaking sessions into existing courses. The present intervention used approximately 15 min sessions twice per week, suggesting that relatively manageable amounts of supplementary practice can be integrated without substantially increasing classroom time. Teachers should also provide learners with clear interaction and feedback guidelines. For example, learners may first be encouraged to complete a conversational turn without interruption and then request focused feedback on selected aspects such as pronunciation, vocabulary, grammatical accuracy, or communicative clarity, rather than receiving excessive correction after every utterance. For learners experiencing relatively high speaking anxiety, individual interaction with GenAI may initially provide a lower-pressure environment in which they can rehearse ideas, repeat responses, and control the pace of interaction before participating in more socially demanding peer or classroom speaking activities. Thus, AI-mediated informal digital learning should be viewed as a structured supplement to, rather than a replacement for, human interaction in EFL speaking instruction.

### Limitations and Future Directions

Several limitations should be considered when interpreting the findings. First, the participants constituted a convenience sample from a single Chinese university, and individual random assignment was not employed. The findings should therefore not be generalized uncritically to other learner populations or educational contexts. Second, although the comparison condition involved speaking practice of comparable frequency and duration, the two conditions differed in interaction partner and feedback environment; consequently, the observed group differences cannot be attributed exclusively to GenAI itself. Third, speaking anxiety and enjoyment were measured through self-report questionnaires, making it difficult to completely exclude expectancy, novelty, or social-desirability effects. The speaking enjoyment measure was also adapted to the speaking context, and future research could further examine its construct validity using item-level analyses. Fourth, although SDT provides a useful interpretive lens, autonomy, competence, and relatedness were not directly measured; therefore, the present findings should not be interpreted as direct empirical evidence for the psychological mechanisms proposed by SDT. Finally, the 10-week delayed post-test provides evidence concerning medium-term retention but cannot establish long-term effectiveness. Future research should employ larger and more diverse samples, longer follow-up periods, multimethod measures, and research designs capable of more clearly separating the effects of AI-mediated interaction from those of practice intensity, feedback, and reduced social-evaluative pressure. An additional methodological consideration concerns the final random-effects specification. During model selection, random-intercept models were compared with models additionally allowing participant-specific random slopes for time using AIC and BIC. Across the three outcomes, the random-intercept models showed lower AIC and BIC values and were therefore retained. Thus, although random slopes were evaluated during model selection, they were not included in the final reported models. Consequently, the final models do not explicitly capture between-participant heterogeneity in rates of change across measurement occasions. Future studies with larger longitudinal samples may further examine the robustness of alternative random-effects structures. A methodological consideration is that separate mixed-effects models were estimated for speaking ability, speaking anxiety, and speaking enjoyment, and an additional correction for multiple testing across outcomes was not applied. This decision was based on the theoretical distinction among the three constructs and the objective of examining intervention effects on each outcome independently. Applying a highly conservative correction across conceptually different outcomes may increase the probability of Type II errors and reduce sensitivity to meaningful educational effects. Nevertheless, future studies with larger samples may consider multivariate mixed-effects approaches or prespecified false discovery rate procedures to provide additional control over multiple outcome analyses.

The present results suggest some directions for subsequent research. Basically, longitudinal research is needed to track whether reducing speaking anxiety and increasing speaking enjoyment, as well as the capability offered by GenAI, can be maintained over extended teaching periods. Moreover, the current research concentrated on voice mode, though future research can study the multimodal incorporation of GenAI with other tools, like video, to investigate the way enhanced immersion influences the fulfillment of psychological needs. Subsequent research needs to take a comparative method to investigate various student profiles to specify the way personal differences in enjoyment or anxiety interplay with GenAI efficacy. Finally, research needs to concentrate on the repetitive co-design of AI tools, guaranteeing that the development of GenAI characteristics is informed by the particular emotional and educational needs of EFL students. These efforts can guarantee that GenAI acts as a strong support for psychological concepts and linguistic development in language teaching.

## Figures and Tables

**Table 1:** Internal Consistency Reliability of the Study Instruments.

Instrument	Pretest α	Post-Test α	Delayed Post-Test α
Speaking enjoyment questionnaire	0.86	0.89	0.88
Speaking anxiety questionnaire	0.92	0.93	0.92

Note. α = Cronbach’s alpha.

**Table 2:** Descriptive Statistics and Baseline Equivalence at Pretest.

Variable	Group	*n*	M	SD	*t*	*p*
Speaking ability	EG	45	10.84	1.97	0.42	.677
	CG	44	10.66	2.05		
Speaking anxiety	EG	45	60.11	7.54	−0.38	.706
	CG	44	60.68	6.77		
Speaking enjoyment	EG	45	15.47	2.44	0.29	.774
	CG	44	15.32	2.39		

Note. Speaking Ability scores were based on the FCE Speaking Test (0–20).

**Table 3:** Means and Standard Deviations Across the Three Measurement Occasions.

Variable	Group	Pretest M (SD)	Post-Test M (SD)	Delayed Post-Test M (SD)
Speaking ability	EG	10.84 (1.97)	15.92 (1.83)	15.31 (1.79)
	CG	10.66 (2.05)	11.74 (1.91)	11.48 (1.95)
Speaking anxiety	EG	60.11 (7.54)	45.38 (6.41)	46.57 (6.62)
	CG	60.68 (6.77)	57.92 (6.43)	58.46 (6.58)
Speaking enjoyment	EG	15.47 (2.44)	21.16 (1.91)	20.54 (1.98)
	CG	15.32 (2.39)	16.43 (2.21)	16.21 (2.27)

**Table 4:** Linear Mixed-Effects Model Predicting Speaking Ability.

Fixed Effect	B	SE	*t*	*p*	95% CI
Intercept	10.66	0.29	36.76	<.001	[10.09, 11.23]
Group (Experimental)	0.18	0.41	0.44	.662	[−0.63, 0.99]
Time (Post-test)	1.08	0.24	4.50	<.001	[0.61, 1.55]
Time (Delayed)	0.82	0.25	3.28	.001	[0.33, 1.31]
Group × Post-test	4.00	0.34	11.76	<.001	[3.33, 4.67]
Group × Delayed	3.65	0.36	10.14	<.001	[2.94, 4.36]

**Table 5:** Linear Mixed-Effects Model Predicting Speaking Anxiety.

Fixed Effect	B	SE	*t*	*p*	95% CI
Intercept	60.68	1.02	59.49	<.001	[58.68, 62.68]
Group (Experimental)	−0.57	1.44	−0.40	.690	[−3.39, 2.25]
Time (Post-test)	−2.76	0.82	−3.37	.001	[−4.37, −1.15]
Time (Delayed)	−2.22	0.84	−2.64	.009	[−3.87, −0.57]
Group × Post-test	−11.97	1.16	−10.32	<.001	[−14.25, −9.69]
Group × Delayed	−11.32	1.21	−9.36	<.001	[−13.69, −8.95]

**Table 6:** Linear Mixed-Effects Model Predicting Speaking Enjoyment.

Fixed Effect	B	SE	*t*	*p*	95% CI
Intercept	15.32	0.36	42.56	<.001	[14.61, 16.03]
Group (Experimental)	0.15	0.51	0.29	.771	[−0.85, 1.15]
Time (Post-test)	1.11	0.29	3.83	<.001	[0.54, 1.68]
Time (Delayed)	0.89	0.31	2.87	.005	[0.28, 1.50]
Group × Post-test	4.58	0.41	11.17	<.001	[3.77, 5.39]
Group × Delayed	4.18	0.43	9.72	<.001	[3.34, 5.02]

**Table 7:** Bonferroni-Adjusted Pairwise Comparisons.

Variable	Comparison	Mean Difference	*p*
Speaking ability	Pretest–Post-test	5.08	<.001
	Pretest–Delayed	4.47	<.001
	Post-test–Delayed	0.61	.072
Speaking anxiety	Pretest–Post-test	14.73	<.001
	Pretest–Delayed	13.54	<.001
	Post-test–Delayed	1.19	.181
Speaking enjoyment	Pretest–Post-test	5.69	<.001
	Pretest–Delayed	5.07	<.001
	Post-test–Delayed	0.62	.094

**Table 8:** Standardized Between-Group Effect Sizes (Hedges’ *g*).

Outcome	Post-Test *g*	Delayed Post-Test *g*	Interpretation
Speaking ability	2.22	2.02	Very large
Speaking anxiety	−1.97	−1.80	Very large
Speaking enjoyment	2.28	2.02	Very large

Note. Positive values indicate higher scores for the experimental group, whereas negative values for speaking anxiety indicate lower anxiety relative to the control group. Effect sizes are interpreted according to conventional benchmarks (0.20 = small, 0.50 = medium, 0.80 = large); all observed effects exceeded the threshold for a large effect.

## Data Availability

The datasets generated and analyzed during the current study are available from the corresponding author upon reasonable request. The data are not publicly available due to privacy and ethical considerations concerning human participants.
